# Evaluation of mass dog vaccination campaign in Techiman, Ghana

**DOI:** 10.1371/journal.pntd.0014080

**Published:** 2026-10-05

**Authors:** Prince Kyere Dwaah, Sylvia Afriyie Squire, Nana Yaa Awua-Boateng, Denis Dekugmen Yar, David Kando, Abraham Num, Princess Amissah, Helen Djang-Fordjour

**Affiliations:** 1 Veterinary Service Directorate, Disease Investigation Farm/Regional Veterinary Laboratory, Techiman, Bono East, Ghana; 2 University of Skill Training and Entrepreneurial Development, Faculty of Environment and Public Health Education, Public Health Department, Asante-Mampong, Ghana; 3 Animal Health Division, Council for Scientific and Industrial Research (CSIR) - Animal Research Institute, Accra, Ghana; 4 Department of Animal Resources Development, CSIR College of Science and Technology, Accra, Ghana; 5 Ghana Health Service, Municipal Health Directorate, Techiman, Bono East, Ghana; 6 Ghana Health Service, Adisadel Urban Health Centre, Cape Coast, Central Region, Ghana; 7 Sunyani Technical University, Faculty of Applied Science and Technology, Department of Agriculture, Sunyani, Ghana; 8 Kwame Nkrumah University of Science and Technology, Faculty of Agriculture, College of Agriculture and Natural Resources, Department of Animal Science, Kumasi, Ghana; Universitetet i Oslo, NORWAY

## Abstract

**Background:**

Across Sub-Saharan Africa, implementation of mass dog vaccination programmes is frequently constrained by inconsistent vaccine procurement, financing limitations, cold-chain requirements, and weaknesses in distribution systems, rather than complete vaccine unavailability. Achieving and sustaining at least 70% vaccination coverage in dog populations is widely recognized as essential for interrupting transmission; however, there is limited operational evidence from municipal campaigns conducted in rapidly urbanizing, resource-constrained settings. We evaluated the implementation and outcomes of a One Health-coordinated free mass dog rabies vaccination campaign conducted in September 2024 in Techiman South Metropolitan, Ghana.

**Methodology:**

A mixed-methods post-campaign assessment was undertaken two weeks after campaign completion. Quantitative data were collected from 200 dog-owning households using structured questionnaires, and administrative vaccination records were reviewed to estimate overall coverage. In addition, key informant interviews were conducted with veterinary officers, public health professionals, environmental health personnel, and community leaders involved in campaign delivery. Descriptive statistics were used to summarize household participation and awareness indicators, while qualitative data were analyzed thematically.

**Principal findings:**

Among surveyed households, 59.5% owned more than one dog and 51.5% reported vaccinating at least one dog during the campaign. Awareness that rabies is fatal increased from 45% (self-reported pre-campaign recall) to 75% after the campaign period, while awareness that rabies is preventable increased from 40% to 70%. Administrative records indicated that approximately 5,600 dogs were vaccinated, corresponding to an estimated 70% metropolitan-level coverage based on projected dog population figures. Participants identified strong multisectoral collaboration and World Rabies Day mobilization as key facilitators. Implementation barriers included vaccine supply delays, limited cold-chain capacity, inadequate access for peri-urban communities, and the absence of strategies to vaccinate free-roaming dogs.

**Conclusions/Significance:**

The evaluation demonstrated that the Techiman mass dog vaccination campaign achieved administrative vaccination coverage exceeding the internationally recommended threshold while identifying implementation barriers that may influence effective population immunity. The findings highlight the importance of improving vaccine logistics, expanding access to peri-urban communities, strengthening monitoring systems, and incorporating strategies for vaccinating free-roaming dogs during future campaigns.

## Author summary

Rabies is almost always fatal once symptoms begin, yet it can be prevented through vaccination of dogs, which are responsible for nearly all human infections in Africa. Global strategies call for vaccinating at least 70% of dogs each year to stop transmission, but there is limited evidence on how well this works in fast-growing African cities with limited resources. We evaluated a free mass dog rabies vaccination campaign carried out in Techiman, Ghana, using a One Health approach that brought together veterinary services, public health authorities, and community leaders. We interviewed 200 dog-owning households and reviewed official vaccination records. We also spoke with frontline staff involved in the campaign. Official records suggested that roughly three-quarters of the estimated dog population was vaccinated, meeting the recommended coverage threshold. Many households reported improved knowledge about rabies following the campaign. However, not all dog-owning households participated, and free-roaming dogs were not systematically vaccinated. Logistical challenges, including vaccine supply delays and limited access in peri-urban communities, were also identified. Our findings show that coordinated municipal campaigns can reach high vaccination numbers even in resource-limited settings. At the same time, accurate monitoring and inclusion of free-roaming dogs are essential to ensure that reported coverage reflects true protection. Strengthening these components will help Ghana and similar countries move closer to eliminating dog-mediated human rabies.

## Introduction

Rabies is an acute viral zoonosis that remains endemic, mostly in many parts of Africa and Asia. It is estimated that 59,000 human deaths occur annually worldwide, with more than 95% attributable to dog-mediated transmission [1,2]. Despite the availability of safe and effective vaccines for both humans and animals, rabies continues to impose a substantial public health burden in low- and middle-income countries (LMICs), where access to post-exposure prophylaxis (PEP), surveillance capacity, and sustained animal vaccination programs are often limited [3,4]. Children and underserved populations are disproportionately affected, reflecting inequities in health care access, dog ownership practices, and coverage of animal health services [1].

From an epidemiological perspective, sustained interruption of dog-mediated rabies transmission is achievable through high annual vaccination coverage in domestic dog populations. Both mathematical modelling and empirical field studies indicate that vaccinating at least 70% of the dog population can interrupt transmission and reduce spillover into human populations, even in settings with high dog density and substantial free-roaming dog populations [5,6]. Mass dog vaccination interrupts transmission of the rabies virus at its animal source by reducing infection among reservoir dog populations. In contrast, post-exposure prophylaxis protects exposed individuals after a potentially rabid animal bite by preventing disease progression before symptom onset [7,8].

Despite this evidence base, implementing mass dog vaccination at scale in LMICs remains challenging. Common barriers include incomplete estimates of the dog population, limited veterinary workforce capacity, cold-chain and vaccine supply constraints, heterogeneous community participation, and difficulties in accessing peri-urban and free-roaming dog populations [3,9]. Reported vaccination coverage therefore often relies on administrative data that do not fully account for unregistered or free-roaming dogs and may consequently overestimate true population-level protection. These challenges highlight the importance of independent post-campaign evaluations that combine household-level surveys with qualitative assessments of program implementation [9,10].

In response to persistent global rabies transmission, the World Health Organization, together with the Food and Agriculture Organization, the World Organisation for Animal Health, and UNICEF, launched the “Zero by 30” strategic framework to eliminate human deaths from dog-mediated rabies by 2030 [6]. Central to this framework is a One Health approach that integrates veterinary services, public health systems, and community engagement to strengthen vaccination delivery, surveillance, and awareness. While national-level progress has been documented in selected regions, evidence from subnational and municipal vaccination campaigns particularly in rapidly urbanizing, resource-limited settings remains limited [7].

Ghana continues to experience endemic rabies transmission, with recurrent reports of dog bites and sporadic human rabies cases across multiple regions [11,12]. Dog vaccination coverage remains variable and is frequently constrained by financial barriers, limited enforcement of dog ownership regulations, and low public awareness [12,13]. Techiman South Municipality, a rapidly growing commercial and transport hub with high human–dog contact rates in the Bono East Region, has been identified as a recurrent hotspot for dog bites and confirmed human rabies cases, reflecting the interaction of dense human populations, free-roaming dogs, and constrained veterinary infrastructure [13,14].

Addressing these operational barriers, which include incomplete vaccination coverage, logistical constraints, and difficulties reaching free-roaming dogs, underscores the need for systematic evaluation of campaign implementation. A One Health – coordinated free mass dog rabies vaccination campaign was implemented in Techiman South Municipality in September 2024, aligned with World Rabies Day activities. The campaign sought to improve access to vaccination services, increase public awareness, and achieve vaccination coverage consistent with WHO recommendations. Understanding the operational performance of such campaigns is essential for informing future rabies control strategies, particularly in urban and peri-urban LMIC settings where program sustainability and equitable coverage remain critical challenges, according to WHO (2023), Del Rio Vilas et al. [7] and Mbilo et al. [9].

The objective of this study was to evaluate the vaccination coverage, community reach, and implementation challenges of the Techiman mass dog rabies vaccination campaign using a mixed-methods post-campaign assessment. By combining household survey data, administrative vaccination records, and qualitative insights from key stakeholders, this study provides operational information, which refers to how campaign activities were delivered, including logistics, coordination, vaccine distribution, community mobilization, and service delivery whiles implementation-level evidence refers to evidence describing the extent to which the intervention reached the intended population, barriers encountered during delivery, and factors influencing successful implementation in comparable urban LMIC settings.

## Materials and methods

### Ethics statement

This study was conducted as an operational evaluation of a routine government-led rabies vaccination campaign rather than biomedical or clinical research involving human participants. No biological samples were collected, no experimental intervention was introduced, and all information was obtained anonymously following written informed consent. Administrative approval to conduct the study was granted by the Bono East Regional Veterinary Office, the Techiman Metropolitan Veterinary Services Directorate, and the Techiman Metropolitan Health Directorate with the Approval No.: VSD/OH-RES/2024/017. Confidentiality of participants was maintained throughout the study by removing personal identifiers before analysis.

### Study area

The study was conducted in Techiman South Metropolitan, located in the Bono East Region of Ghana. Techiman is a rapidly urbanizing commercial and transport hub with an estimated population of approximately 206,865 residents [15]. The metropolitan comprises urban, peri-urban, and adjoining rural communities and supports substantial free-roaming and owned dog populations. Recurrent reports of dog bites and confirmed human rabies cases have been documented in the municipality, indicating a sustained risk of rabies transmission [13,14].

### Description of the One Health Rabies vaccination campaign

The vaccination campaign was implemented using One Health principles through coordinated collaboration between veterinary, public health, environmental health, and community sectors, which was implemented in September 2024 in Techiman South Metropolis. Veterinary teams were responsible for vaccine delivery, cold-chain management, dog handling, and vaccination, while public health personnel supported risk communication, community sensitization, and linkage of dog-bite victims to appropriate health facilities for post-exposure prophylaxis. Community leaders and local media facilitated mobilization, dissemination of campaign messages, and identification of vaccination sites. Campaign activities were aligned with World Rabies Day, leveraging global messaging to enhance public trust and participation. This multisectoral collaboration aimed to simultaneously reduce rabies risk at the animal source, improve community awareness of rabies prevention, and strengthen coordination between animal and human health services. We consistently describe the campaign as being implemented using One Health principles.

### Study design

This study employed a cross-sectional mixed-methods implementation study design conducted approximately two weeks after completion of the September 2024 metropolitan mass dog rabies vaccination campaign. The study was designed to assess campaign implementation, community reach, vaccination coverage, and implementation barriers using complementary quantitative and qualitative approaches.

The quantitative component consisted of a household survey among dog-owning households to describe campaign participation, exposure to campaign communication activities, and respondents’ awareness and knowledge of rabies. Administrative vaccination records maintained by the Veterinary Services Directorate were reviewed to estimate campaign reach and administrative vaccination coverage.

The qualitative component involved key informant interviews with personnel directly involved in campaign implementation to explore operational experiences, intersectoral coordination, factors that facilitated implementation, and barriers that affected campaign delivery.

Because no baseline survey had been conducted before the campaign, respondents were asked to recall selected pre-campaign awareness indicators. These retrospective responses were collected solely to provide contextual information and were not treated as baseline measurements. Consequently, comparisons between recalled pre-campaign and post-campaign responses were interpreted descriptively, and no causal inference regarding campaign effectiveness was made. This approach acknowledges the possibility of recall bias while providing additional context for interpreting post-campaign findings.

### Study population

The study population comprised adult dog owners residing in communities where the September 2024 mass dog rabies vaccination campaign was implemented in the Techiman South Metropolitan Area, Bono East Region, Ghana. Eligible participants were individuals aged 18 years or older who had owned at least one dog during the campaign period, had lived in the study area for a minimum of six months before the campaign, and were available to provide informed consent. Restricting the study to dog-owning households ensured that respondents had direct experience with the vaccination campaign and were able to provide reliable information on household participation, exposure to campaign communication activities, and perceptions of campaign implementation.

For the qualitative component, participants were purposively selected from individuals directly involved in planning, coordinating, or implementing the vaccination campaign. These included veterinary officers, public health professionals, environmental health officers, and community leaders. Their participation provided diverse perspectives on campaign implementation, intersectoral collaboration, operational facilitators, and barriers encountered during programme delivery.

### Sampling strategy

#### Quantitative component.

A multistage non-probability sampling strategy was employed. First, communities that participated in the metropolitan rabies vaccination campaign were purposively selected to ensure inclusion of areas where the intervention had been implemented. Purposive selection was appropriate because the objective of the study was to assess campaign implementation rather than estimate population prevalence.

Within the selected communities, eligible dog-owning households were pre-screened for dog ownership before enrolment. Since no comprehensive sampling frame of dog-owning households existed within the municipality, snowball sampling was used to identify additional eligible households. One adult (≥18 years) respondent was interviewed from each household.

The minimum sample size was determined using Cochran’s formula [16] for estimating a single population proportion $n=\frac{Z^{\text{2}}P(\text{1}-P)}{d^{\text{2}}}$, where: n = required sample size, Z = standard normal deviate corresponding to the 95% confidence level (1.96), P = anticipated household participation proportion (0.50) and d = margin of error (0.07).

A prevalence estimate of 50% was selected because no previous metropolitan-level household survey had estimated participation in mass dog vaccination campaigns under comparable conditions in the study area. This conservative assumption yields the largest possible sample size when prior estimates are unavailable. A precision level of 7% was considered acceptable because the study primarily sought to describe campaign implementation rather than estimate prevalence with high precision, while remaining feasible within available logistical and financial resources.

The calculated minimum sample size was 196 households. To ensure a complete dataset and compensate for possible incomplete questionnaires, the target sample size was increased to 200 households.

#### Qualitative component.

Purposive sampling was used to recruit key informants directly involved in planning, coordinating, supervising, or implementing the vaccination campaign. Ten participants were interviewed, comprising three veterinary officers, two public health professionals, two environmental health officers, and three community leaders.

The objective of the qualitative component was to obtain complementary implementation perspectives across sectors rather than to compare professional groups individually. Recruitment continued until no substantially new implementation issues emerged across interviews, indicating adequate implementation-level thematic saturation. Because the study focused on understanding campaign implementation rather than generating profession-specific themes, thematic saturation was considered across the collective dataset rather than within each stakeholder subgroup [17].

### Data collection

#### Household survey.

Quantitative data were collected using a structured interviewer-administered questionnaire developed from published rabies vaccination and One Health implementation literature and adapted to the local context. Before field implementation, the questionnaire was reviewed by experts in veterinary public health and piloted among 30 dog-owning households in neighbouring municipalities (Techiman North, Wenchi, and Sunyani West) to assess clarity, relevance, sequencing of questions, and respondent comprehension. Data obtained during the pilot study were used solely to refine the questionnaire and were not included in the final analysis.

The final questionnaire collected information on household demographic characteristics, dog ownership characteristics, exposure to campaign communication activities, participation in the vaccination campaign, awareness of the campaign, knowledge of rabies transmission and prevention and perceived accessibility of vaccination services.

In this study, awareness was defined as respondents’ prior exposure to information regarding rabies or the vaccination campaign, whereas knowledge referred to respondents’ factual understanding of rabies transmission, prevention, fatality, and control. These operational definitions were applied consistently throughout data collection and analysis.

#### Key informant interviews.

Semi-structured key informant interviews were conducted with personnel directly, including ten key informant interviews completed with three veterinary officers, two public health officials, two environmental health officers and three community leaders involved in campaign implementation. An interview guide was developed to explore experiences relating to campaign coordination, multisectoral collaboration, community engagement, logistical arrangements, operational facilitators, and implementation barriers.

Interviews were conducted face-to-face by trained researchers in private settings convenient for participants. With participants’ permission, all interviews were audio-recorded and supplemented by field notes documenting contextual observations and non-verbal information.

#### Administrative vaccination records.

Administrative records were obtained from the Techiman Veterinary Services Directorate, including reports from the Metropolitan Veterinary Clinic and the Disease Investigation Farm/Regional Veterinary Laboratory. These records documented the total number of dogs vaccinated during the September 2024 campaign.

Administrative vaccination coverage was estimated by comparing the total number of vaccinated dogs with the projected municipal dog population derived from the most recent veterinary population estimates using the WHO–recommended human-to-dog ratio of 5:1. Because no recent census of the owned and free-roaming dog population was available, this estimate represents administrative vaccination coverage rather than a direct measure of population immunity. The administrative records did not distinguish between owned and free-roaming dogs, and vaccination status of free-roaming dogs was not routinely documented. Consequently, the reported coverage should be interpreted as an operational indicator of campaign reach rather than confirmation that the WHO-recommended 70% population immunity threshold was achieved.

## Results

### Household dog ownership and vaccination participation

A total of 200 dog-owning households participated in the survey. Of these, 81 (40.5%) owned one dog, while 119 (59.5%) owned two or more dogs. Overall, 103 households (51.5%) reported vaccinating at least one dog during the September 2024 mass dog rabies vaccination campaign, whereas 97 (48.5%) reported that none of their dogs was vaccinated (Table 1).

**Table 1:** Household dog ownership and participation in the 2024 mass dog rabies vaccination campaign among surveyed households in Techiman, Ghana (N = 200).

Household characteristic	Category	Frequency (n)	Percentage (%)
Number of dogs owned	One dog	81	40.5
	Two or more dogs	119	59.5
Household participation in the vaccination campaign	Vaccinated at least one dog	103	51.5
	Did not vaccinate any dog	97	48.5

Values represent the distribution of surveyed dog-owning households. Household participation was defined as vaccination of at least one owned dog during the September 2024 mass dog rabies vaccination campaign.

Among households that participated in vaccination, the number of dogs vaccinated per household varied according to dog ownership size. Households owning multiple dogs were more likely to report partial vaccination of their dogs than complete household-level coverage.

### Community awareness of rabies

Exposure to campaign communication increased substantially following the vaccination campaign (Table 2). Before the campaign, based on participant recall, 76 (38.0%) respondents reported having heard about the vaccination campaign, compared with 190 (95.0%) after the campaign. Radio and television, community networks, and health workers were the most frequently reported sources of campaign information.

**Table 2:** Campaign awareness and rabies knowledge factors reported by dog-owning households before (participant recall) and after the 2024 mass dog rabies vaccination campaign in Techiman, Ghana (N = 200).

Domain	Factor Assessed	Pre-campaign (participant recall) n (%)	Post-campaign n (%)
Campaign awareness	Had heard about the rabies vaccination campaign	76 (38.0)	190 (95.0)
	Primary source of campaign information: Radio/television	70 (35.0)	76 (38.0)
	Primary source of campaign information: Health workers	56 (28.0)	64 (32.0)
	Primary source of campaign information: Community networks (family, friends, community meetings)	74 (37.0)	90 (45.0)
Rabies knowledge	Correctly identified rabies as a fatal disease once clinical signs appear	90 (45.0)	150 (75.0)
	Correctly recognised that rabies can be prevented through vaccination	80 (40.0)	140 (70.0)

1 *Campaign awareness* was defined as respondents’ previous exposure to information about the mass dog rabies vaccination campaign or its communication activities.

2 *Rabies knowledge* was defined as respondents’ correct understanding of rabies transmission, prevention, and fatality.

3 Pre-campaign responses were obtained retrospectively from participants and are presented for contextual purposes only. They should not be interpreted as true baseline measurements because they may be subject to recall bias.

4 Percentages are based on the total sample of 200 respondents.

Respondents’ rabies knowledge also improved. The proportion of respondents who correctly recognised rabies as a fatal disease increased from 90 (45.0%) before the campaign to 150 (75.0%) after the campaign. Similarly, the proportion correctly identifying vaccination as an effective means of preventing rabies increased from 80 (40.0%) to 140 (70.0%) (Table 2). Because pre-campaign responses were obtained retrospectively, these comparisons are descriptive and should not be interpreted as evidence of causal effects attributable to the campaign.

### Implementation barriers and enabling factors identified through key informant interviews

Thematic analysis identified four recurrent implementation themes that were consistently supported through triangulation of interview data, household survey findings, and administrative records (Table 3). While community engagement and multisectoral collaboration were discussed across all stakeholder groups, some operational themes were more strongly emphasised by particular participants. Vaccine supply delays and cold-chain constraints were primarily described by veterinary personnel but were corroborated by public health professionals and community leaders. Accessibility challenges affecting peri-urban communities were consistently reported by environmental health officers, community leaders, and public health personnel. Similarly, the absence of structured approaches for vaccinating free-roaming dogs was recognised across multiple stakeholder groups as an important limitation affecting effective population-level vaccination coverage.

**Table 3:** Triangulated qualitative themes, supporting quantitative and administrative findings, and illustrative quotations from key informant interviews during the 2024 mass dog rabies vaccination campaign in Techiman, Ghana.

Theme	Subtheme	Supporting quantitative/ administrative findings	Illustrative quotation	Interpretation
Community engagement and multisectoral collaboration	Collaboration among veterinary, public health, environmental health, local authorities and community leaders	Administrative records showed successful implementation across participating communities with 5,600 of an estimated 7,568 dogs vaccinated (74.0%). Most households reported exposure to campaign communication activities.	“The collaboration between veterinary staff, environmental health officers, and community leaders made mobilisation much easier. Each institution had a defined role, which reduced duplication of effort and improved coordination during the campaign.” (Veterinary Officer 2)	Multisectoral coordination facilitated community mobilisation and efficient campaign delivery.
Community awareness and communication	Radio announcements, community information centres, churches, mosques, schools and interpersonal communication	Household survey showed campaign awareness increased from 38.0% (participant recall) before implementation to 95.0% after the campaign.	“Many dog owners came because they heard repeated announcements on the local radio and information centre. Without those announcements, many people would not have known the campaign was taking place.” (Community Leader 3)	Multiple communication channels substantially improved campaign awareness and promoted participation.
	Unequal dissemination of campaign information	Some households reported non-participation despite high awareness.	“Some remote communities only became aware of the campaign after the vaccination team had already completed its activities. Earlier mobilisation would have improved participation in those areas.” (Environmental Health Officer 2)	Delayed communication reduced participation in some communities despite overall high awareness.
Operational barriers to campaign delivery	Delayed vaccine supply	Household participation (51.5%) was lower than administrative vaccination coverage (74.0%).	“We experienced delays in receiving vaccines on the first day, which affected our schedule. Some community members waited for several hours, while others returned home before their dogs could be vaccinated.” (Veterinary Officer 1)	Delays in vaccine availability reduced the efficiency of campaign implementation.
	Inadequate cold-chain equipment and transport	Administrative records confirmed campaign implementation, but interviews highlighted logistical constraints.	“Cold boxes and transport were not always adequate for the number of communities we had to cover. This limited the number of vaccination points we could operate simultaneously.” (Veterinary Officer 3)	Logistical limitations constrained timely delivery of vaccination services.
	Long travel distances and waiting time	Household respondents cited accessibility as a reason for non-participation.	“People living far from the vaccination centres wanted to participate, but the distance and transport costs discouraged many from bringing their dogs.” (Community Leader 2)	Physical accessibility remained an important implementation barrier.
Vaccination of free-roaming dogs	Absence of structured vaccination strategy	Administrative coverage reflected only dogs presented for vaccination and did not account for free-roaming dogs.	“Our campaign was successful for owned dogs, but we had no organised approach for capturing and vaccinating free-roaming dogs. Those animals remained outside the programme.” (Veterinary Officer 4)	Administrative coverage may overestimate effective population immunity because free-roaming dogs were largely excluded.
	Recommendations for future campaigns	Key informants recommended mobile vaccination teams and Catch–Vaccinate–Release approaches.	“Future campaigns should combine fixed vaccination points with mobile outreach and organised vaccination of free-roaming dogs. That would increase coverage and strengthen rabies prevention.” (Public Health Officer 2)	Future campaigns should strengthen implementation by incorporating targeted strategies for vaccinating free-roaming dogs.

**Abbreviation:** KII, Key Informant Interview*.*

### Administrative vaccination data and estimated coverage

Administrative records from the Techiman Metropolitan Veterinary Services Directorate showed that the September 2024 mass dog rabies vaccination campaign achieved substantial coverage across the intervention area. A total of 5,600 out of an estimated 8,000 dogs were vaccinated during the campaign, corresponding to an administrative vaccination coverage of 70.0% (Table 4). Based on projected dog population estimates derived from municipal veterinary reports and application of the WHO – recommended human-to-dog ratio of 5:1, this corresponds to an estimated vaccination coverage of approximately 70% at the metropolitan level.

**Table 4:** Administrative campaign indicators for the 2024 mass dog rabies vaccination campaign in Techiman, Ghana.

Indicator	Value
Estimated dog population	8,000
Dogs vaccinated	5,600
Administrative vaccination coverage	70.0%
Vaccination centres	6
Campaign duration	7 days (1 week)

Because this estimate relied on projected dog population figures and routine administrative records, it should be interpreted as an indicator of campaign reach rather than direct evidence that the WHO-recommended herd immunity threshold was achieved. The estimate did not account for uncertainties in the projected dog population or for free-roaming dogs that were largely outside the vaccination programme.

## Discussion

This implementation study evaluated the delivery and outcomes of a municipal mass dog rabies vaccination campaign conducted in Techiman, Ghana. By combining household survey data, administrative vaccination records, and key informant interviews, the study generated complementary evidence on campaign reach, community participation, and implementation barriers. Administrative records indicated that 5,600 out of an estimated 8,000 dogs (70.0%) were vaccinated during the campaign, while household-level findings and qualitative data identified important operational constraints that may have influenced effective population coverage. Collectively, these findings provide practical evidence to inform the planning and implementation of future municipal rabies vaccination campaigns in comparable resource-limited settings.

Administrative records indicated an estimated vaccination coverage of 70.0%, which was equal to the minimum 70% threshold recommended by the WHO for interrupting dog-mediated rabies transmission [6]. Nevertheless, this estimate should be interpreted cautiously because it was derived from projected dog population estimates and routine vaccination records that did not distinguish between owned and free-roaming dogs. Consequently, the reported administrative coverage reflects campaign reach rather than direct measurement of population immunity. Similar concerns have been reported in other rabies-endemic settings, where reliance on administrative data alone may overestimate effective vaccination coverage because free-roaming dogs and inaccuracies in dog population estimates are not adequately captured [5,9].

The household survey demonstrated that approximately half of the surveyed households reported vaccinating at least one dog during the campaign. Although household participation appeared lower than the administrative coverage estimates, the two measures represent different aspects of campaign performance. Administrative coverage describes the overall number of dogs vaccinated relative to the estimated dog population, whereas household participation reflects whether individual households presented at least one dog for vaccination. Differences between these measures have been documented in previous studies and emphasise the value of combining administrative records with household surveys to obtain a more comprehensive understanding of campaign implementation and community participation [3,9].

Respondents reported higher levels of campaign awareness and rabies knowledge following the vaccination campaign than before the campaign. Because information describing the pre-campaign period was obtained retrospectively through participant recall, these findings should be interpreted as descriptive rather than causal. Nevertheless, the results suggest that the communication activities implemented alongside the vaccination campaign successfully reached many dog-owning households. Radio and television, community networks, and health workers were the principal channels through which respondents received campaign information, highlighting the importance of using multiple communication platforms to improve community engagement. Similar communication approaches have been associated with increased participation in rabies prevention activities in other African settings [11,12].

The qualitative findings complemented the quantitative results by identifying both enabling factors and implementation barriers that influenced campaign delivery. Key informants consistently described multisectoral collaboration among the Veterinary Services Directorate, Ghana Health Service, Environmental Health Unit, local authorities, and community leaders as an important enabling factor that facilitated community mobilisation and coordinated service delivery. These observations are consistent with international recommendations that advocate collaboration across the animal health, human health, and community sectors to strengthen rabies prevention programmes [6,7]. However, this study evaluated the implementation of a vaccination campaign delivered using One Health principles and did not evaluate the effectiveness of the One Health approach itself.

Several implementation barriers were also identified. Delays in vaccine supply, inadequate cold-chain capacity, long waiting times at vaccination points, and limited accessibility of vaccination services for peri-urban communities reduced opportunities for some households to participate. These operational barriers have been widely documented in rabies-endemic countries and continue to constrain the effectiveness and sustainability of mass dog vaccination programmes [3,9]. Addressing these barriers through improved logistical planning, timely vaccine procurement, expansion of vaccination sites, and strengthened cold-chain infrastructure could improve campaign efficiency and accessibility in future vaccination rounds.

An important implementation barrier identified by both household respondents and key informants was the absence of structured strategies for vaccinating free-roaming dogs. Administrative vaccination activities primarily targeted owned dogs brought voluntarily to vaccination points, leaving many free-roaming dogs outside the campaign. Because free-roaming dogs may contribute to sustained rabies transmission in endemic communities, their exclusion could reduce effective population-level immunity despite achieving high administrative vaccination coverage. Previous studies have demonstrated that incorporating targeted approaches, such as catch – vaccinate – release programmes, can substantially improve vaccination coverage among free-roaming dog populations and strengthen rabies control efforts [5,6].

A major strength of this study was the integration of three complementary data sources – household surveys, administrative vaccination records, and qualitative interviews – which enabled triangulation of findings and provided a broader understanding of campaign implementation than would have been possible using a single method. Household surveys described community participation and campaign awareness, administrative records quantified campaign reach, and key informant interviews provided contextual explanations for the observed quantitative findings. This mixed-methods approach strengthened the interpretation of the results and generated practical implementation evidence relevant to municipal rabies control programmes.

This study has several limitations that should be considered when interpreting the findings. First, the cross-sectional design precluded causal inference regarding changes in rabies awareness and knowledge. Second, pre-campaign information was obtained retrospectively and may therefore be subject to recall bias. Third, administrative vaccination coverage relied on projected dog population estimates derived from the WHO-recommended human-to-dog ratio and did not include systematic enumeration or vaccination of free-roaming dogs, thereby limiting the precision of coverage estimates. Fourth, participants in the household survey were restricted to adult dog owners because they were considered the principal household decision-makers regarding dog ownership and vaccination. Consequently, the study did not capture children’s knowledge, exposure patterns, or bite-reporting behaviour, despite children representing one of the populations most vulnerable to dog-mediated rabies. Finally, purposive community selection and snowball sampling may limit the generalisability of the findings beyond dog-owning households in the study area.

Overall, the findings demonstrate that the 2024 Techiman mass dog rabies vaccination campaign achieved substantial administrative vaccination coverage and successfully engaged many dog-owning households through coordinated multisectoral implementation. At the same time, the study identified important implementation barriers that should be addressed to improve future campaigns. Strengthening operational planning, expanding access to underserved communities, improving vaccination monitoring systems, and incorporating structured approaches for vaccinating free-roaming dogs could enhance the effectiveness of future municipal rabies vaccination campaigns and contribute to sustained progress towards the elimination of dog-mediated human rabies in Ghana.

## Conclusion

This implementation study demonstrates that the 2024 Techiman mass dog rabies vaccination campaign successfully reached a substantial proportion of the estimated owned dog population through coordinated implementation using One Health principles. Administrative records indicated that approximately 5,600 dogs were vaccinated, corresponding to an estimated administrative vaccination coverage of 70.0% based on projected municipal dog population estimates. Household findings further demonstrated high community awareness of the campaign and improved rabies knowledge among participating dog-owning households, while qualitative evidence highlighted the value of multisectoral collaboration in supporting campaign delivery.

However, the reported administrative coverage should not be interpreted as direct evidence that the WHO-recommended herd immunity threshold was achieved. Uncertainty surrounding projected dog population estimates, together with the absence of structured strategies for vaccinating free-roaming dogs, may have reduced effective population-level immunity. Future campaigns should therefore strengthen dog population estimation, incorporate affordable post-campaign monitoring systems, expand vaccination strategies for free-roaming dogs through approaches such as Catch–Vaccinate–Release, and improve access to underserved communities. These measures would increase confidence in vaccination coverage estimates and strengthen progress towards the elimination of dog-mediated human rabies in Ghana.

## Supporting information

S1 File Questionnaire (Household questionnaire used for the quantitative survey). (DOCX)

S2 File Semi-structured key informant interview guide. (DOCX)

S1 Appendix Mapping of interview questions to study themes. (DOCX)
