# Supplementary material for: Evaluation of mass dog vaccination campaign in Techiman, Ghana
Source: PLoS Negl Trop Dis. 2026 Oct 5;20(10):e0014080. doi: 10.1371/journal.pntd.0014080 (PMC13638042; doi:10.1371/journal.pntd.0014080)
Supplement: Supplementary file 2 [file pntd.0014080.s001.docx]

**SI File: QUESTIONNAIRE**

**Title:** *Field Assessment of the Mass Rabies Vaccination Campaign in Techiman*

**Section 1: Demographic Information**

1. Age:	☐ Under 18	☐ 18-30	☐ 31-45	☐ 46-60	☐ Over 60

2. Gender:	☐ Male	☐ Female	☐ Prefer not to say

3. Occupation: ☐ Farmer	☐ Trader	☐ Public Service Worker

☐ Student	☐ Other (please specify): ………………………

4. Level of Education: ☐ No formal education	☐ Primary	☐ Secondary

☐ Tertiary	☐ Vocational training

5. Household Size:	☐ 1-3 members	☐ 4-6 members	☐ More than six members

**Section 2: Dog Ownership and Vaccination**

6. Do you own any dogs? ☐ Yes	☐ No (If No, skip to Section 4)

7. How many dogs do you own? ☐ 1	☐ 2-3	☐ More than 3

8. Why do you own a dog(s)?	☐ Security	☐ Companionship

☐ Herding livestock	☐ Other (please specify): ………………………………

9. Were your dogs vaccinated during the free rabies vaccination campaign?

☐ Yes	☐ No (If No, skip to Question 11)

10. How many of your dogs were vaccinated during the campaign?

☐ 1	☐ 2-3	☐ More than 3

11. If your dogs were not vaccinated, what was the reason?

☐ Did not know about the campaign	☐ Could not bring my dog to the vaccination site

☐ Vaccine shortage	☐ Other (please specify): ……………….

12. Have your dog been vaccinated for rabies before this campaign?

☐ Yes	☐ No	☐ I do not know

**Section 3: Awareness and Knowledge of Rabies**

13. were you aware of rabies and its dangers before the vaccination campaign?

☐ Yes	☐ No

14. How did you first hear about rabies?

☐ Health worker	☐ Radio/TV	☐ Family/Friend

☐ Community Meeting	☐ Other (please specify): ………………………..

15. What is the primary way rabies is transmitted to humans?

☐ Dog bites	☐ Drinking contaminated water

☐ Contact with animal blood	☐ I do not know

16. Do you know that rabies is preventable through vaccination? ☐ Yes	☐ No

17. Are you aware that rabies is fatal if not treated promptly? ☐ Yes	☐ No

18. Where would you go if a dog bit you or someone in your family?

☐ Hospital/clinic	☐ Traditional healer	☐ Do nothing	☐ I do not know

**Section 4: Participation in the Rabies Vaccination Campaign**

19. How did you hear about the free rabies vaccination campaign?

☐ Radio/TV announcement	☐ Local health workers	☐ Community Meeting

☐ Friends/Family	☐ Other (please specify): ……………………………

20. Did you attend the World Rabies Day event in Techiman? ☐ Yes	☐ No

21. If yes, what motivated you to participate in the vaccination campaign?

☐ Concern for my dog's health	☐ Fear of rabies

☐ It was free	☐ Other (please specify): ……………………….

22. Was the vaccination process easy and accessible? ☐ Yes	☐ No

23. If No, what difficulties did you experience?

☐ Long waiting time	☐ Location was too far

☐ Not enough staff	☐ Other (please specify): …………………….

24. Do you feel that the campaign was well-organized? ☐ Yes	☐ No

25. Do you think such campaigns should be held regularly? ☐ Yes	☐ No

**Section 5: General Feedback**

26. How likely are you to get your dogs vaccinated again next year?

☐ Very Likely	☐ Somewhat likely

☐ Unlikely	☐ I don't know

27. Would you recommend this vaccination campaign to others in your community?

☐ Yes	☐ No

28. What could be improved in future rabies vaccination campaigns?

☐ More awareness/advertisement	☐ More vaccination sites

☐ Faster service	☐ Other (please specify): ……………

29. Do you have any other comments or suggestions about the rabies vaccination campaign?

……………………………………………………………………………………………………………………………………………………………………………………………………

Thank you for your participation!
