# Supplementary material for: Evaluation of mass dog vaccination campaign in Techiman, Ghana
Source: PLoS Negl Trop Dis. 2026 Oct 5;20(10):e0014080. doi: 10.1371/journal.pntd.0014080 (PMC13638042; doi:10.1371/journal.pntd.0014080)
Supplement: Supplementary file 3 [file pntd.0014080.s002.docx]

**S2 File:** *Semi-Structured Key Informant Interview Guide*

**Study Title:** *Mass Dog Rabies Vaccination Campaign in Techiman, Ghana:*

**Introduction**

Good morning/afternoon.

Thank you for agreeing to participate in this interview. We are conducting this study to understand the implementation of the 2024 mass dog rabies vaccination campaign in Techiman. We are interested in learning about your experiences, the factors that facilitated the campaign, challenges encountered, and recommendations for improving future campaigns.

There are no right or wrong answers. We are interested in your personal experiences and opinions. The interview will take approximately *30 – 45 minutes*. With your permission, the discussion will be audio-recorded to ensure that your responses are accurately captured. All information will remain confidential, and your identity will not be disclosed in any report or publication.

1. Do you agree to participate?	□ Yes	□ No
2. Permission to audio-record?	□ Yes	□ No

**Participant Information**

**Participant ID:**	…………………………………………….

**Date:**	…………………………………………….

**Location:**	…………………………………………….

**Institution:**

□ Veterinary Services Directorate	□ Ghana Health Service

□ Environmental Health Unit	□ Metropolitan Assembly

□ Traditional Authority	□ Community Leader

□ Other ……………………………………………………………………………….

**Position/Role:**	………………………………………………...

**Years involved in rabies control:**	………………………………………………...

**Interviewer:**	………………………………………………...

**Section A:** Role in the Vaccination Campaign

**A1.**	*Please describe your role during the September 2024 mass dog rabies vaccination campaign.*

**Probes**:	Planning/ Community mobilisation/ Vaccine distribution/

Vaccination/ Supervision/ Data reporting

**A2.**	*How long have you been involved in rabies prevention or vaccination programmes?*

………………………………………………………………………………………….

**Section B:** Campaign Planning and Coordination

**B1.**	*How was the vaccination campaign planned before implementation?*

**Probes**	Planning meetings/ Stakeholder involvement/ Resource mobilisation/ Logistics

**B2.**	*Which organisations participated in the campaign?*

**Probes**	Veterinary Services/ Ghana Health Service/ Environmental Health/

Assembly/ NGOs/ Community leaders

**B3.**	*How effective was collaboration among these organisations?*

**Probes**	Communication/ Coordination/ Decision-making/ Shared responsibilities

**Section C:** Community Awareness and Participation

**C1.** *How was information about the vaccination campaign communicated to the public?*

**Probes**	Radio/ Information centres/ Churches/ Mosques

Schools/ Social media/ Community volunteers

**C2.**	*Which communication methods worked best?* *Why?*

…………………………………………………………………………………………………

**C3.**	*What factors encouraged households to participate?*

**Probes**	Free vaccination/ Community trust/ Awareness/ Accessibility

**C4.**	*What factors discouraged participation?*

**Probes**:	Distance/ Waiting time/ Fear of dogs/ Lack of awareness/ Cost of transport

**Section D:** Campaign Implementation

**D1.**	*Overall, how would you describe the implementation of the campaign?*

…………………………………………………………………………………………………

**D2.**	*What aspects of implementation worked particularly well?*

**Probes**: Teamwork/ Vaccine availability/ Community response/ Logistics

**D3.**	*What operational barriers affected campaign implementation?*

**Probes**: Vaccine supply/ Cold chain/ Staffing/ Transport/ Equipment/ Weather/ Funding

**D4.**	*How were these challenges managed?*

…………………………………………………………………………………………………

**Section E:** Vaccination Coverage

**E1.** *In your opinion, why was the campaign able (or unable) to achieve high vaccination coverage?*

…………………………………………………………………………………………………

**E2.** *Were there any communities that were difficult to reach?* *Why?*

…………………………………………………………………………………………………

**E3.** *Did all owned dogs have equal access to vaccination?* *Explain*.

…………………………………………………………………………………………………

**Section F:** Free-Roaming Dogs

**F1.** *How were free-roaming dogs managed during the campaign?*

…………………………………………………………………………………………………

**F2.** *Do you think free-roaming dogs represent an important challenge for rabies control? Why?*

…………………………………………………………………………………………………

**F3.** *What strategies would improve vaccination of free-roaming dogs?*

**Probes**: Catch–Vaccinate–Release/ Mobile vaccination teams/ Community volunteers/ Dog registration

**Section G:** Community Awareness and Rabies Knowledge

**G1.** *Based on your experience, how knowledgeable were community members about rabies before the campaign?*

…………………………………………………………………………………………………

**G2.** *Did awareness change after the campaign? Please explain.*

…………………………………………………………………………………………………

**G3.** *What misconceptions about rabies did you commonly encounter?*

…………………………………………………………………………………………………

**Section H:** Sustainability

**H1.** *What should be maintained in future campaigns?*

…………………………………………………………………………………………………

**H2.** *What should be improved?*

…………………………………………………………………………………………………

**H3.** *What additional resources would improve implementation?*

…………………………………………………………………………………………………

**H4.** *How can future campaigns become more sustainable?*

…………………………………………………………………………………………………

**Section I:** Lessons Learned

**I1.** *What were the most important lessons learned from this campaign?*

…………………………………………………………………………………………………

**I2.** *If another municipality wanted to organise a similar campaign, what advice would you give?*

…………………………………………………………………………………………………

**I3.** *Is there anything else you would like to add?*

…………………………………………………………………………………………………

**Interview Closing:** *Thank you very much for your participation. Your responses will contribute to improving rabies prevention and control programmes in Ghana.*
