# Supplementary material for: Evaluation of mass dog vaccination campaign in Techiman, Ghana
Source: PLoS Negl Trop Dis. 2026 Oct 5;20(10):e0014080. doi: 10.1371/journal.pntd.0014080 (PMC13638042; doi:10.1371/journal.pntd.0014080)
Supplement: Supplementary file 4 [file pntd.0014080.s003.docx]

**S1 Appendix:** *Mapping of Interview Questions to Study Themes*

| Interview Domain | Expected Theme | Linked Study Objective |
| --- | --- | --- |
| Campaign planning and coordination | Community engagement and multisectoral collaboration | Describe implementation processes |
| Communication and mobilisation | Community awareness and communication | Assess campaign awareness and participation |
| Operational experiences | Operational barriers to campaign delivery | Identify implementation barriers and facilitators |
| Free-roaming dogs | Vaccination of free-roaming dogs | Explore barriers affecting vaccination coverage |
| Lessons learned | Recommendations for future implementation | Inform future rabies vaccination programmes |
