# Supplementary material for: Evaluation of mass dog vaccination campaign in Techiman, Ghana
Source: PLoS Negl Trop Dis. 2026 Oct 5;20(10):e0014080. doi: 10.1371/journal.pntd.0014080 (PMC13638042; doi:10.1371/journal.pntd.0014080)
Supplement: Supplementary file 5 [file pntd.0014080.s005.docx]

**Reviewer Comments**

Lines 1 & 2: suggested title – Evaluation of mass dog vaccination campaign in Techiman, Ghana

Lines 54-57: what is stated here is a bit vague, as neither it declares the ethical conduct of the study, nor it justifies the reasons for conducting the study without ethical approval.

Line 92: Check the “S” in “Sub-Sahara” and be consistent. In Sub-Saharan Africa, one of the bottlenecks to mass dog vaccination is unavailability of vaccines. Make sure what is stated here is with scientific evidence.

Lines 110 and 143: are awareness and knowledge used interchangeably? If that is the case, it needs correction, as awareness is a broader concept. Check this issue throughout the manuscript and address. Again, another confusion appeared on line 272.

Line 114: better to replace the word “stakeholders” with “participants” or use a specific description of the subject.

Line 116: when authors use “reported challenges”, what do they really want to state? If these challenges were barriers, clearly state as barriers that hindered the success of the campaign, as the use of the term challenge makes it vague.

Lines 120-126 (conclusion): it is a bit over-stretched and concluded on contents that are not found within this study. For example, the study has not considered any hypothesis or method that could support the success of “short-duration” campaign. It makes this an ambitious statement. To make such a conclusion needs a comparative study. But generally, such evidence has been reported in other studies elsewhere. The conclusion is expected to clearly show what has been achieved by the study (as per the objectives set before) and what does that finding mean with respect to its public health, economic or rabies control perspectives. Hence, avoid use of what is stated in previous reports or evidence, rather focus on what the findings of this study tell us. With this respect, please **restate** the conclusion section. There is a well-written statement on line 147-148. Conclude in that way.

Line 128: I will add “Techiman” as a keyword.

Line 167: the 59,000 estimated annual deaths are at the global level, not limited to Africa and Asia.

Lines 180-182: needs rewording, as mass dog vaccination and PEP are applied at different stages in rabies control. Mass dog vaccination works more on breaking the transmission of rabies from dogs to humans (more of preventive role), while PEP works to stop the development of rabies after an individual is exposed to rabies virus.

Line 213: what does “these” refer to?

Lines 217-220: needs citation.

Line 223: the word “integrating” could be replaced by other words such as using, aggregating or …

Line 225: operational and implementation-level evidence – authors should make sure that the two concepts are dealt explicitly.

Line 245: better to use the latest report, as such statistical reports are made available annually.

Line 252: Not clear for readers when framework is used. Is that to mean OH principles? If not, it needs to be revised.

Line 265: seems there are multiple OH approaches. Needs rewording.

Lines 266-280: there is a mix of study design and data collection tools here. The study design is cross-sectional and it used mixed-methods approach. For the “study design” section, this information is sufficient. The rest should be stated in other sections or subsections.

Line 267: the use of “evaluation” here misleads the readers. Evaluation vs study?

Line 269-270: what do authors mean when stating “limited pre-campaign collected retrospectively”? It needs a detailed explanation on the how and why it was collected; as well as on its methodological correctness.

Lines 271-275: if pre-campaign assessment of awareness was not made, how could the use of recall-based items in the questionnaire address that? Either it should be evidence supported using citations or this part need to be excluded.

Line 279-277: for purpose of clarity and flow, “exposure to campaign communication activities” should come before “household participation in vaccination”.

Line 281: usually, study population comes before study design, immediately after study area (or after “Description of the One Health Rabies Vaccination Campaign”, in the case of this manuscript).

Line 286: what does “campaign implementation coverage” here? If it is referring to the scope or reach of the campaign, it needs to be stated that way.

Lines 287-289: the sampling method used and the purpose stated there need scientific evidence support (citations) so that applicability could be justified.

Lines 290-294: what was the assumption regarding the size of the population, finite or infinite?

The use of 50% vaccination proportion needs to be explained. Are the authors sure that there is no previous study or report on this condition under similar or closely related settings. The use of 7% margin of error needs a clear explanation, as the conventional is 5%. The use of 7% margin of error reduces the sample size. If sample size is reduced intentionally in an abused way, its representativeness will be questioned. If the target population is assumed finite, there are adjusted formulae to manage that. What is the base to add only 4 households, to account for incomplete responses? This is also another serious matter.

Line 301: it is always good considering 30 and above participants for pre-testing a questionnaire but compared to the overall sample size of this study, this maybe large. And whether individuals interviewed from these 30 households are included in the final sample size or not need to be stated.

Lines 312-313: check for its correctness.

Lines 324-325: what do authors mean when stating campaign facilitators and implementation barriers? It is not clear here.

Lines 325-326: the phrase “triangulated during interpretation” is ambiguous. Needs a clear description.

Line 327: The ethics section needs further explanation on its feasibility and scientific support, making sure that administrative permissions might be equally treated as ethical approvals. Otherwise, this is really questionable section.

Line 353: better to write the description and refer the table or figure as (Table 1) or (Figure 1).

Lines 359-365: table 1 and figure 1 can be merged. Presentation needs to be improved.

Lines 368-371: needs rewording and paraphrasing to minimize redundancy. The context that the word awareness is not clear here too.

Line 377: Table 2 shows knowledge and awareness. What is knowledge? And what is awareness? These should be clearly stated in the methods section. In addition, which item questions were used to assess knowledge, and which ones were used to assess awareness? We usually use the term “variable” in the methods section, as statistical term. In the results section, it can be substituted by factor.

Line 382: write as 5,600 out of 8,000 …

Line 390: better to give it a heading title that describes the findings (themes). One suggestion is barriers and enablers of the campaign.

Lines 391-392: these could be moved to the methods section.

Line 409: The table title needs to be self-explanatory and sentence case. What is WRD? Better to use the full form when it is a title. Quotes need to be written within “…” and in italics.

General comment on the results section

- The results section is very shallow, does not indicate any form of triangulation of quantitative and qualitative findings.
- Results presentation needs improvement, like scientific lines (three rows for tables), quotations from KIIs.
- The administrative data record has been covered only with descriptions, better to include in table and indicate the figures.
- There is a mix of some statements that are expected to be stated in the methods section.

Line 427: the methods used are not clear. Implementation, evaluation, operational, one health. This needs to be addressed. It can be seen that the mass dog vaccination intervention has followed One Health approach. And the study can be an implementation type of research. But it is difficult to state that the study has applied One Health approach. Authors need to address this throughout.

Line 432: the vaccination coverage of 75%. Under lines 113 and 386, it is indicated as 74%. Which one is the correct?

Line 465-466: the absence of strategies to vaccinate free-roaming dogs, is described as limitation, challenge, barrier … authors need to get the right use and be consistent.

Lines 472-479: the study lacks the power to state such findings, as it is an evaluation study of a One Health mass dog vaccination program/intervention. It is difficult to declare that the study employed a One Health approach.

Line 486: operational vs implementation? Authors need to address this throughout the document.

487-490: based on the data and findings of the study, these statements are over-stretched and beyond the scope of the study. Needs rewriting, aligning the descriptions with the current findings.

Line 491: conclusion (lines 492-514) – needs rewriting, based on current data and findings. In the conclusion, one expects the answers to the research question/objectives and the implications. Hence, make the conclusion specific to questions, data and findings, with clear implications of the findings.

Generally

- There is a lack of methods to address the stated objectives.
- The results section is very shallow. The qualitative part is less addressed, quotes are expected in the results section. Where is the triangulation of quantitative and qualitative data? As the themes are expected to be formed from subthemes and/or codes, inclusion of some quotes, especially together with quantitative results makes the triangulation clear.
- The discussion is shallow and lacks coherence. In the discussion section, one expects a brief introductory paragraph of main findings; following the order of findings in this introduction, comparing the findings with previous findings, showing public health or rabies control implications, its economic impact, and the like. All paragraphs should be structured that way.
- Conclusion needs revision. It should focus on the findings to the objectives and should be precise.
- Authors are advised to follow a scientific paper writing. In addition, interpretations should be aligned to the existing data, no need to over-stretch the findings.
