# Supplementary material for: Evaluation of mass dog vaccination campaign in Techiman, Ghana
Source: PLoS Negl Trop Dis. 2026 Oct 5;20(10):e0014080. doi: 10.1371/journal.pntd.0014080 (PMC13638042; doi:10.1371/journal.pntd.0014080)
Supplement: Supplementary file 6 [file pntd.0014080.s007.docx]

**MINISTRY OF FOOD AND AGRICULTURE**

**DISEASE INVESTIGATION FARM AND VETERINARY LABORATORY**

**BONO EAST REGION TECHIMAN**

**TEL**: +233(0) 200759509	Post Office Box 122

**Mobile:** +233(0) 540622111	Techiman – Bono East

**E-mail:** pkdwaah@aamusted.edu.gh

**Our Ref No:** DIF/TCH/REC01/26

**Your Ref No: …………………..**	July 12, 2026

The Editor-in-Chief
*PLOS Neglected Tropical Diseases*

Dear Editor-in-Chief,

On behalf of all the authors, we sincerely thank you and the reviewer for the careful evaluation of our manuscript and for the constructive comments and suggestions. We greatly appreciate the time and effort invested in reviewing our work. The comments have been invaluable in improving the scientific quality, methodological rigour, clarity, and overall presentation of the manuscript.

We have carefully considered every comment and have revised the manuscript accordingly. All modifications have been incorporated into the revised manuscript, with changes clearly indicated in the tracked-changes version. Below, we provide a detailed, point-by-point response to each comment and describe how the manuscript has been revised.

**Response to Reviewer**

**Comment 1:** *Suggested title "Evaluation of mass dog vaccination campaign in Techiman, Ghana."*

**Response:** Thank you for this valuable suggestion. We agreed that the original title overemphasized the One Health concept rather than the principal focus of the study. The title has therefore been revised to improve clarity and better reflect the study objective.

**Revision made:** The title has been changed to*: "Evaluation of a Mass Dog Rabies Vaccination Campaign in Techiman, Ghana."*

**Comment 2:** *The ethics statement is vague, and it neither declares ethical conduct nor justifies conducting the study without ethical approval.*

**Response:** We appreciate this important observation. The Ethics Statement has been substantially revised to comply with the requirements of PLOS Neglected Tropical Diseases. We now clarify that the study was an operational evaluation of an ongoing public health intervention that did not alter routine service delivery or involve experimental procedures. Administrative authorization was obtained from the relevant implementing institutions before data collection, and all participants provided written informed consent. The revised manuscript also explicitly distinguishes administrative permission from institutional ethical approval and discusses the rationale for the study design.

**Revision made:** The Ethics Statement has been completely rewritten.

**Comment 3:** *Use consistent terminology for "sub-Saharan Africa" and support the statement regarding vaccine availability with scientific evidence.*

**Response:** Thank you for identifying this inconsistency. The terminology has been standardized throughout the manuscript as "sub-Saharan Africa." The statement regarding vaccine availability has also been revised to reflect documented evidence showing that rabies control programmes in the region are constrained by inconsistent vaccine supply, procurement challenges, financing limitations, and cold-chain capacity rather than complete vaccine unavailability. Appropriate references have been added.

**Comment 4:** *Awareness and knowledge appear to be used interchangeably.*

**Response:** We appreciate this methodological observation. Throughout the manuscript, awareness and knowledge have now been clearly distinguished. Awareness refers to respondents having heard about rabies or the vaccination campaign, whereas knowledge refers to respondents' understanding of rabies transmission, prevention, clinical outcome, and control. The Methods section now defines both variables explicitly, and consistent terminology has been applied throughout the Abstract, Results, Discussion, Tables, and Figures.

**Comment 5:** *Replace "stakeholders" with "participants" or a more specific term.*

**Response:** We agree. The term "stakeholders" has been replaced throughout the manuscript with more specific descriptions such as "key informants," "participants," "veterinary officers," or "community leaders," depending on the context.

**Comment 6:** *Clarify whether "reported challenges" refers to implementation barriers.*

**Response:** Thank you for this suggestion. We have replaced the phrase "reported challenges" with "implementation barriers" wherever appropriate to improve precision and consistency.

**Comment 7:** *The conclusion overstates findings beyond the study design.*

**Response:** We appreciate this important comment. The conclusion has been completely rewritten. Statements implying causal relationships or superiority of short-duration campaigns have been removed. The revised conclusion now focuses exclusively on findings generated by this evaluation, including vaccination coverage, campaign reach, implementation barriers, and implications for future rabies control programmes.

**Comment 8:** *Add "Techiman" as a keyword.*

**Response:** Done. "Techiman" has been included among the keywords.

**Comment 9:** *The estimated 59,000 annual rabies deaths are global, not limited to Africa and Asia.*

**Response:** We appreciate this correction. The statement has been revised to indicate that the estimate represents the global burden of rabies.

**Comment 10:** *Clarify the distinction between mass dog vaccination and post-exposure prophylaxis (PEP).*

**Response:** We agree. The Introduction has been revised to explain that mass dog vaccination interrupts rabies transmission at the animal source, whereas PEP prevents disease development following human exposure.

**Comment 11:** *Clarify the word "these."*

**Response:**
The sentence has been revised to identify the specific subject being referenced.

**Comment 12:** *Provide supporting citations.*

**Response:** Appropriate references have been added to support the statements identified by the reviewer.

**Comment 13:** *Replace "integrating."*

**Response:** The wording has been revised. The manuscript now refers to "combining" household survey findings, administrative records, and qualitative interviews.

**Comment 14:** *Differentiate operational evidence from implementation evidence.*

**Response:** The manuscript now clearly distinguishes operational evidence (campaign logistics and delivery processes) from implementation evidence (how the intervention functioned in practice).

**Comment 15:** *Use the most recent statistical report.*

**Response:** The outdated reference has been replaced with the most recent available report.

**Comment 16**

**Clarify the meaning of "framework."**

**Response:**
The manuscript now refers to "One Health principles" rather than a "One Health framework" and explains how multisectoral collaboration was implemented during campaign delivery.

**Comment 17**

**Reword statements implying multiple One Health approaches.**

**Response:**
The relevant sentences have been revised for clarity and consistency.

**Comment 18**

**Separate study design from data collection methods.**

**Response:**
The Materials and Methods section has been reorganized into clearly defined subsections, including Study Design, Study Population, Sampling, Data Collection, Administrative Records, Data Analysis, and Ethical Considerations.

**Comment 19**

**Clarify the use of "evaluation."**

**Response:**
The terminology has been standardized throughout the manuscript. The study is consistently described as a mixed-methods cross-sectional evaluation.

**Comment 20**

**Explain retrospective pre-campaign information.**

**Response:**
Additional methodological details have been provided describing how recall-based information was collected, its purpose, and its limitations. Recall bias has also been acknowledged in the limitations section.

**Comment 21**

**Provide evidence supporting recall-based assessment or remove it.**

**Response:**
Supporting methodological references have been included, and the manuscript explicitly states that these data were descriptive and not intended to estimate causal change.

**Comment 22**

**Improve paragraph flow.**

**Response:**
The paragraph has been reorganized to improve logical flow.

**Comment 23**

**Move Study Population before Study Design.**

**Response:**
The manuscript has been reorganized accordingly.

**Comment 24**

**Clarify campaign implementation coverage.**

**Response:**
The phrase has been replaced with "campaign geographic reach" to improve clarity.

**Comment 25**

**Provide citations supporting the sampling approach.**

**Response:**
Relevant methodological references have been incorporated.

**Comment 26**

**Improve sample size justification.**

**Response:**
The sample size calculation has been substantially revised. We now justify the use of a 50% expected proportion, explain the selected precision level, clarify the population assumptions, and provide an improved justification for the final sample size.

**Comment 27**

**Clarify pilot testing.**

**Response:**
The manuscript now states that the pilot participants were excluded from the final study sample.

**Comment 28**

**Correct methodological statements.**

**Response:**
The identified section has been revised for accuracy.

**Comment 29**

**Clarify facilitators, barriers, and triangulation.**

**Response:**
Definitions have been added, and the triangulation process has been described in greater methodological detail. The Results section has also been expanded to better integrate qualitative and quantitative findings.

**Comment 30**

**Strengthen the ethics section.**

**Response:**
The Ethics section has been rewritten to clearly distinguish administrative authorization from institutional ethical approval and to explain the ethical safeguards implemented during the study.

**General Comments**

We appreciate the reviewer's broader observations regarding the Methods, Results, Discussion, and Conclusion. In response, we have undertaken a comprehensive revision of the manuscript. Major improvements include:

- clarification of the study objectives and terminology;
- substantial restructuring of the Materials and Methods section;
- improved justification of the sampling strategy and sample size;
- clearer differentiation between awareness and knowledge;
- expanded qualitative analysis with illustrative quotations from key informants;
- explicit triangulation of quantitative, qualitative, and administrative data;
- improved presentation of tables and figures in accordance with scientific reporting standards;
- strengthened Discussion with clearer interpretation of findings in relation to previous literature;
- updated references using the most recent evidence;
- rewritten Conclusion that aligns directly with the study objectives and findings without extending beyond the available evidence.

We believe these revisions have considerably strengthened the manuscript and addressed all concerns raised by the reviewer. We are grateful for the insightful comments, which have significantly improved the quality and clarity of our work.

Thank you for your careful consideration of our revised manuscript.

Yours faithfully,

Prince Kyere Dwaah (Corresponding Author),
**Email**: [pkdwaah@aamusted.edu.gh](mailto:pkdwaah@aamusted.edu.gh),

**ORCID**: 0009-0009-9515-3213.

**On behalf of all co-authors**
