# Supplementary material for: Evaluation of mass dog vaccination campaign in Techiman, Ghana
Source: PLoS Negl Trop Dis. 2026 Oct 5;20(10):e0014080. doi: 10.1371/journal.pntd.0014080 (PMC13638042; doi:10.1371/journal.pntd.0014080)
Supplement: Supplementary file 7 [file pntd.0014080.s008.docx]

**Response to the Editor**

**Manuscript Title:** *Evaluation of Mass Dog Vaccination Campaign in Techiman, Ghana*

Dear Editor,

We sincerely thank you for your careful assessment of our manuscript and for your constructive comments. We appreciate your recognition that our study contributes valuable operational evidence on One Health rabies control in Africa. We have carefully considered every comment and have revised the manuscript accordingly. Our detailed responses are presented below.

**Comment 1: Estimates of Dog Population**

**Editor Comment**

*The reliance on a projected baseline of 8,000 dogs using the WHO recommendation of 5:1 leaves the stated 74% coverage metric vulnerable to denominator inflation. Given that the study explicitly notes a lack of strategies for free-roaming dogs, the true population-level immunity may be lower. It would be interesting to hear the authors' perspective on how future iterations of this campaign might realistically integrate low-cost, resource-limited monitoring tools to validate these population projections and ensure the 70% WHO herd immunity threshold is truly met.*

**Response**

We thank the Editor for this insightful observation and fully agree that administrative vaccination coverage derived from projected dog populations should not be interpreted as a direct estimate of population immunity.

As correctly noted, our estimate was based on the WHO-recommended human-to-dog ratio because no recent municipal dog census was available. We have now strengthened the manuscript by explicitly emphasizing that the reported coverage represents administrative vaccination coverage rather than verified herd immunity, particularly because free-roaming dogs were largely excluded from vaccination activities. This clarification is consistent with our original description of the administrative records and their limitations.

Following the Editor's recommendation, we have substantially expanded the Discussion to describe feasible monitoring approaches that can be implemented in resource-constrained municipalities. Specifically, we now recommend:

i. periodic community-based dog population surveys;
ii. household-based post-vaccination coverage assessments;
iii. photographic mark-resight techniques for estimating free-roaming dog populations;
iv. mobile phone-based vaccination registers with GPS-enabled vaccination points;
v. community volunteer reporting systems; and
vi. integration of Catch-Vaccinate-Release (CVR) programmes with simple post-campaign monitoring.

These low-cost approaches would improve estimation of the true dog population, permit validation of administrative coverage estimates, and provide a more reliable assessment of whether the WHO-recommended 70% vaccination threshold has been achieved.

**Revision made**

The Discussion and Conclusion sections have been revised to clearly distinguish administrative coverage from population immunity and to include practical recommendations for affordable monitoring systems suitable for municipal rabies programmes.

**Comment 2: Household Sampling**

**Editor Comment**

*Could the authors clarify their sampling design? Did these 200 households represent a general population sample or were participants pre-screened for dog ownership?*

**Response**

We appreciate this request for clarification.

The 200 households were not intended to represent the general population of Techiman South Metropolitan. Rather, they comprised dog-owning households only, because the primary objective of the quantitative survey was to evaluate campaign participation, vaccination uptake, awareness, and perceptions among households that were eligible to participate in the vaccination campaign.

Eligible households were therefore pre-screened for dog ownership before enrolment. Communities where the campaign had been implemented were purposively selected, after which eligible dog-owning households were identified through snowball sampling because no sampling frame of dog-owning households existed within the municipality. One eligible adult respondent was interviewed from each household. This sampling strategy is already described in the Methods section but has now been clarified further to prevent possible misunderstanding.

We have also revised the Results section to consistently refer to dog-owning households rather than households generally, thereby avoiding overgeneralisation.

**Revision made**

Additional clarification has been incorporated into the Methods and Results sections explaining that all participants were pre-screened dog owners and that findings should not be interpreted as representative of the entire metropolitan population.

**Comment 3: Key Informant Interviews (KII)**

**Editor Comment**

*The sample spans four distinct sectors. With only 2 - 3 participants per subgroup, was thematic saturation reached within any single sector? Were themes such as Operational Challenges and Inaccessibility reported across all groups or only one?*

**Response**

We thank the Editor for raising this important qualitative methodological issue.

The purpose of the qualitative component was not to compare perspectives across professional groups, but rather to obtain complementary implementation-level insights from stakeholders jointly involved in campaign planning and delivery.

Although the participants represented four sectors, they shared similar responsibilities during implementation and interacted closely throughout the campaign. Consequently, our objective was to achieve overall implementation-level thematic saturation, rather than saturation within each professional subgroup.

To address the Editor's concern, we have revised the Methods to clarify that recruitment continued until no substantially new implementation issues emerged across interviews, consistent with the study objectives.

We have also revised the Results to indicate more explicitly that:

i. **Community engagement and multisectoral collaboration** were discussed across all stakeholder groups.
ii. **Operational challenges**, including vaccine delays and cold-chain constraints, were primarily reported by veterinary personnel but were corroborated by public health professionals and community leaders.
iii. **Accessibility challenges affecting peri-urban communities** were consistently identified by community leaders, environmental health officers, and public health personnel, with veterinary officers acknowledging similar logistical constraints.
iv. The absence of structured approaches for vaccinating free-roaming dogs was recognised across multiple sectors.

Accordingly, the themes presented represent triangulated findings rather than sector-specific conclusions.

**Revision made**

The Methods and Results sections have been revised to clarify the concept of implementation-level saturation and to indicate the stakeholder groups contributing to each theme.

**Comment 4: Age Restriction on Participants**

**Editor Comment**

*Restricting participants to adults is understandable. However, since children are the population most vulnerable to rabies, does this approach obscure important gaps in community risk reduction and bite reporting?*

**Response**

We appreciate this thoughtful observation.

We fully agree that children represent one of the populations at greatest risk of dog-mediated rabies in sub-Saharan Africa. However, the objectives of the present evaluation were to assess campaign implementation, household participation, dog ownership, and vaccination practices. In Ghana, decisions regarding dog ownership, presentation of dogs for vaccination, and household health-seeking behaviour are generally made by adults. Consequently, restricting interviews to adult household representatives was considered appropriate for addressing the study objectives.

Nevertheless, we agree that relying solely on adult respondents may not fully capture children's knowledge, exposure patterns, bite-reporting behaviour, or educational needs.

Accordingly, we have added this issue as an explicit limitation and recommend that future One Health evaluations include school-based surveys, child-focused knowledge assessments, and behavioural studies to better understand rabies risk among children.

**Revision made**

A limitation has been added acknowledging that exclusion of participants younger than 18 years may have limited understanding of rabies knowledge and exposure among children, and recommendations for future research have been included.

**Comment 5: Results**

**Editor Comment**

*Based on the comments above, I recommend the authors reinvestigate their results.*

**Response**

We appreciate this recommendation and have carefully reviewed the Results section in its entirety.

Following this review, we have revised the Results to improve consistency with the study design and avoid overinterpretation. Specifically, we:

i. consistently describe vaccination estimates as administrative vaccination coverage;
ii. clarify that household findings refer exclusively to dog-owning households;
iii. explain that awareness comparisons are descriptive because pre-campaign information was obtained retrospectively; and
iv. clarify the interpretation of qualitative themes and their relationship to the different stakeholder groups.

No changes were required to the numerical findings because they accurately reflect the underlying data.

**Comment 6: Conclusions**

**Editor Comment**

*I would like to see whether the authors consider that their conclusions stand.*

**Response**

We appreciate this recommendation.

Following revision of the Results and Discussion, we also revised the Conclusion to ensure that it remains fully supported by the evidence.

The revised conclusion now avoids implying that the campaign definitively achieved protective herd immunity. Instead, it states that the campaign achieved substantial administrative vaccination coverage while acknowledging that the absence of vaccination strategies for free-roaming dogs and reliance on projected population estimates limit certainty regarding true population-level immunity. The revised conclusion also places greater emphasis on strengthening surveillance, dog population monitoring, and strategies targeting free-roaming dogs in future campaigns. These revisions are consistent with the study's findings and limitations.

**Summary Comment**

**Editor Comment**

*The authors present a review of a valuable One Health mass dog rabies vaccination campaign in Ghana that contributes to building a larger picture of dog rabies in Africa.*

**Response**

We sincerely thank the Editor for this encouraging assessment of our work.

Our objective was to provide operational evidence from a municipal One Health rabies vaccination campaign that could inform future implementation in Ghana and other rabies-endemic African settings. We greatly appreciate the Editor's constructive suggestions, which have substantially strengthened the methodological clarity, interpretation of findings, and practical relevance of the manuscript.

We believe the revised manuscript addresses all concerns raised and is considerably improved as a result.
