# Supplementary material for: Evaluation of mass dog vaccination campaign in Techiman, Ghana
Source: PLoS Negl Trop Dis. 2026 Oct 5;20(10):e0014080. doi: 10.1371/journal.pntd.0014080 (PMC13638042; doi:10.1371/journal.pntd.0014080)
Supplement: Peer review history

# Peer review: Decision Letter 0

**DOI:** 10.1371/journal.pntd.0014080.r001

**Authors:** Song Liang, Simon Rayner

9 Jul 2026

PNTD-D-26-00375

Evaluation of a One Health mass dog rabies vaccination campaign in a resource-limited urban setting: Evidence from Techiman, Ghana

PLOS Neglected Tropical Diseases

Dear Dr. Dwaah,

Thank you for submitting your manuscript to PLOS Neglected Tropical Diseases. After careful consideration, we feel that it has merit but does not fully meet PLOS Neglected Tropical Diseases's publication criteria as it currently stands. Therefore, we invite you to submit a revised version of the manuscript that addresses the points raised during the review process.

Please submit your revised manuscript within by Sep 07 2026 11:59PM. If you will need more time than this to complete your revisions, please reply to this message or contact the journal office at plosntds@plos.org. When you're ready to submit your revision, log on to https://www.editorialmanager.com/pntd/ and select the 'Submissions Needing Revision' folder to locate your manuscript file.

Please include the following items when submitting your revised manuscript:

* A letter that responds to each point raised by the editor and reviewer(s). You should upload this letter as a separate file labeled 'Response to Reviewers'. This file does not need to include responses to any formatting updates and technical items listed in the 'Journal Requirements' section below.

* A marked-up copy of your manuscript that highlights changes made to the original version. You should upload this as a separate file labeled 'Revised Manuscript with Track Changes'.

* An unmarked version of your revised paper without tracked changes. You should upload this as a separate file labeled 'Manuscript'.

If you would like to make changes to your financial disclosure, competing interests statement, or data availability statement, please make these updates within the submission form at the time of resubmission. Guidelines for resubmitting your figure files are available below the reviewer comments at the end of this letter.

As the corresponding author, your ORCID iD is verified in the submission system and will appear in the published article. PLOS supports the use of ORCID, and we encourage all coauthors to register for an ORCID iD and use it as well. Please encourage your coauthors to verify their ORCID iD within the submission system before final acceptance, as unverified ORCID iDs will not appear in the published article. Only the individual author can complete the verification step; PLOS staff cannot verify ORCID iDs on behalf of authors.

We look forward to receiving your revised manuscript.

Kind regards,

Simon Rayner

Academic Editor

PLOS Neglected Tropical Diseases

Song Liang

Section Editor

PLOS Neglected Tropical Diseases

Shaden Kamhawi

co-Editor-in-Chief

PLOS Neglected Tropical Diseases

orcid.org/0000-0003-4304-636XX

Paul Brindley

co-Editor-in-Chief

PLOS Neglected Tropical Diseases

orcid.org/0000-0003-1765-0002

**Additional Editor Comments (if provided):**

Thank you for submitting your manuscript to PLoS NTD

The two reviews have been submitted. one of which is my own. Both reviews seem to raise concerns about the method and i ask that address them in a revised manuscript.

**Journal Requirements:**

If the reviewer comments include a recommendation to cite specific previously published works, please review and evaluate these publications to determine whether they are relevant and should be cited. There is no requirement to cite these works unless the editor has indicated otherwise.

1) Please ensure that the CRediT author contributions listed for every co-author are completed accurately and in full.

At this stage, the following Authors/Authors require contributions: Sylvia Afriyie Squire. Please ensure that the full contributions of each author are acknowledged in the "Add/Edit/Remove Authors" section of our submission form.

The list of CRediT author contributions may be found here: https://journals.plos.org/plosntds/s/authorship#loc-author-contributions

2) Please upload all main figures as separate Figure files in .tif or .eps format. For more information about how to convert and format your figure files please see our guidelines:

https://journals.plos.org/plosntds/s/figures

3) We have noticed that you have given the  Table 2 in the manuscript file but there is no citation for the table in the manuscript.  Please amend your manuscript to include this table noting that tables should not be uploaded as individual files.

4) In the online submission form, you indicated that The data supporting the findings of this study are available from the corresponding author upon reasonable request. Aggregated anonymized data on vaccination coverage is stored at the Disease Investigation farm and Regional Veterinary Laboratory, the Metropolitan Veterinary Office.. All PLOS journals now require all data underlying the findings described in their manuscript to be freely available to other researchers, either

1. In a public repository

2. Within the manuscript itself

3. Uploaded as supplementary information.

This policy applies to all data except where public deposition would breach compliance with the protocol approved by your research ethics board. If your data cannot be made publicly available for ethical or legal reasons (e.g., public availability would compromise patient privacy), please explain your reasons by return email and your exemption request will be escalated to the editor for approval. Your exemption request will be handled independently and will not hold up the peer review process, but will need to be resolved should your manuscript be accepted for publication. One of the Editorial team will then be in touch if there are any issues.

5) Please include a completed 'Competing Interests' statement in the text box when submitting your production task, including any COIs declared by your co-authors, written in full sentences. If you have no competing interests to declare, please state "The authors have declared that no competing interests exist". You may also provide an updated statement via email.

**Reviewers' Comments:**

Reviewer's Responses to Questions

**Key Review Criteria Required for Acceptance?**

As you describe the new analyses required for acceptance, please consider the following:

**Methods**

-Are the objectives of the study clearly articulated with a clear testable hypothesis stated?

-Is the study design appropriate to address the stated objectives?

-Is the population clearly described and appropriate for the hypothesis being tested?

-Is the sample size sufficient to ensure adequate power to address the hypothesis being tested?

-Were correct statistical analysis used to support conclusions?

-Are there concerns about ethical or regulatory requirements being met?

Reviewer #1: comments are indicated in the document attached as "reviewer comments". The objective, sample size, and ethical considerations sections need to be revised. Especially, the sample size determination is not appropriate. The ethical section needs evidence support.

Reviewer #2: Estimates of dog population

The reliance on a projected baseline of 8,000 dogs using the WHO recommendation of 5:1 leaves the stated 74% coverage metric vulnerable to denominator inflation. Given that the study explicitly notes a lack of strategies for free-roaming dogs, the true population-level immunity may be lower.

It would be interesting to hear the authors perspective on on how future iterations of this campaign might realistically integrate low-cost, resource-limited monitoring tools to validate these population projections and ensure the 70% WHO herd immunity threshold is truly met.

Household sampling

A sample size of 200 households is logistically understandable and consistent with similar pilot-scale One Health evaluations in resource-limited urban environments (e.g., Lusaka). Nevertheless, could the authors clarify their sampling design. For example, did these 200 households represent a general population sample or were participants pre-screened for dog ownership, as this might significantly affect the precision of the reported vaccination and ownership metrics.

KII

The authors rely on 10 key informant interviews to evaluate the One Health integration of the campaign. However, while a sample of 10 can achieve saturation in a highly homogeneous group, couldn’t i argue that the sample is somewhat heterogeneous, i.e., spanning four distinct sectors (veterinary, public health, environmental health, and community leadership) with differing institutional goals, vocabularies, and mandates.?

With only 2 to 3 participants per sub-group, was thematic saturation reached within any single sector? Specifically, you state that “. Four recurrent themes emerged from the thematic analysis” but are themes 2 “Operational Challenges During Campaign Implementation” and 3 “Inaccessibility for Rural and Peri-Urban Communities” reported across all foour groups, or only one?

Age restriction on participants

The restriction of household questionnaires to adults >= 18 is standard and logistically sound regarding household decision-making and animal ownership logistics. However, given that children are commonly the most vulnerable demographic to rabies in sub-Saharan Africa it would be interesting to hear the authors thoughts on this – Does relying solely on adult-focused data obscure critical gaps in community risk-reduction and bite reporting in a particularly vulnerable sub group of the population?

**********

**Results**

-Does the analysis presented match the analysis plan?

-Are the results clearly and completely presented?

-Are the figures (Tables, Images) of sufficient quality for clarity?

Reviewer #1: As indicated in the "reviewer comments", this section needs much improvement.

Reviewer #2: based on the comments above, i would recommend the authors reinvestigate their results

**********

**Conclusions**

-Are the conclusions supported by the data presented?

-Are the limitations of analysis clearly described?

-Do the authors discuss how these data can be helpful to advance our understanding of the topic under study?

-Is public health relevance addressed?

Reviewer #1: This section also needs much improvement. The discussion is very shallow. The conclusion is over-stretched, as the findings presented in the results section are not able to support such statements. Public health relevance is less addressed. Detailed comments are indicated in the two documents attached as in (i) main manuscript: through highlighting, and (ii) reviewer comments: line by line list of comments.

Reviewer #2: Similarly, i would like to see whether the authors consider that their conclusions stand

**********

**Editorial and Data Presentation Modifications?**

Use this section for editorial suggestions as well as relatively minor modifications of existing data that would enhance clarity. If the only modifications needed are minor and/or editorial, you may wish to recommend “Minor Revision” or “Accept”.

Reviewer #1: (No Response)

Reviewer #2: (No Response)

**********

**Summary and General Comments**

Use this section to provide overall comments, discuss strengths/weaknesses of the study, novelty, significance, general execution and scholarship. You may also include additional comments for the author, including concerns about dual publication, research ethics, or publication ethics. If requesting major revision, please articulate the new experiments that are needed.

Reviewer #1: Included in my word document "reviewer comments".

Reviewer #2: The authors present a review of a valuable One Health mass dog rabies vaccination campaign of Ghana that contributes to building a larger picture of dog rabies in Africa.

**********

PLOS authors have the option to publish the peer review history of their article (what does this mean?). If published, this will include your full peer review and any attached files.

If you choose “no”, your identity will remain anonymous but your review may still be made public.

**Do you want your identity to be public for this peer review?**  For information about this choice, including consent withdrawal, please see our Privacy Policy.

Reviewer #1: No

Reviewer #2: No

[NOTE: If reviewer comments were submitted as an attachment file, they will be attached to this email and accessible via the submission site. Please log into your account, locate the manuscript record, and check for the action link "View Attachments". If this link does not appear, there are no attachment files.]

**Figure resubmission:**

While revising your submission, we strongly recommend that you use PLOS’s NAAS tool (https://ngplosjournals.pagemajik.ai/artanalysis) to test your figure files. NAAS can convert your figure files to the TIFF file type and meet basic requirements (such as print size, resolution), or provide you with a report on issues that do not meet our requirements and that NAAS cannot fix.

After uploading your figures to PLOS’s NAAS tool - https://ngplosjournals.pagemajik.ai/artanalysis, NAAS will process the files provided and display the results in the "Uploaded Files" section of the page as the processing is complete. If the uploaded figures meet our requirements (or NAAS is able to fix the files to meet our requirements), the figure will be marked as "fixed" above. If NAAS is unable to fix the files, a red "failed" label will appear above. When NAAS has confirmed that the figure files meet our requirements, please download the file via the download option, and include these NAAS processed figure files when submitting your revised manuscript.

**Reproducibility:**

To enhance the reproducibility of your results, we recommend that authors of applicable studies deposit laboratory protocols in protocols.io, where a protocol can be assigned its own identifier (DOI) such that it can be cited independently in the future. Additionally, PLOS ONE offers an option to publish peer-reviewed clinical study protocols. Read more information on sharing protocols at https://plos.org/protocols?utm_medium=editorial-email&utm_source=authorletters&utm_campaign=protocols

Attachment Submitted filename: PNTD-D-26-00375 _ Reviewed.pdf

Attachment Submitted filename: Reviewer Comments.docx

# Author comment: Author response to Decision Letter 1

**DOI:** 10.1371/journal.pntd.0014080.r002

18 Jul 2026

Attachment Submitted filename: Review Letter.docx

# Editor report: Decision Letter 1

**DOI:** 10.1371/journal.pntd.0014080.r003

**Authors:** Simon Rayner, Qu Cheng

6 Aug 2026

PNTD-D-26-00375R1Evaluation of mass dog vaccination campaign in Techiman, GhanaPLOS Neglected Tropical Diseases Dear Dr. Dwaah, Thank you for submitting your manuscript to PLOS Neglected Tropical Diseases. While you have responded to  the first reviewer, you haven't addressed the original comments raised by the second reviewer. Therefore, we invite you to submit a revised version of the manuscript that addresses these additional points. Please submit your revised manuscript by Oct 05 2026 11:59PM. If you will need more time than this to complete your revisions, please reply to this message or contact the journal office at plosntds@plos.org. When you're ready to submit your revision, log on to https://www.editorialmanager.com/pntd/ and select the 'Submissions Needing Revision' folder to locate your manuscript file. Please include the following items when submitting your revised manuscript:* A letter that responds to each point raised by the editor and reviewer(s). You should upload this letter as a separate file labeled 'Response to Reviewers'. This file does not need to include responses to any formatting updates and technical items listed in the 'Journal Requirements' section below.* A marked-up copy of your manuscript that highlights changes made to the original version. You should upload this as a separate file labeled 'Revised Manuscript with Track Changes'.* An unmarked version of your revised paper without tracked changes. You should upload this as a separate file labeled 'Manuscript'. If you would like to make changes to your financial disclosure, competing interests statement, or data availability statement, please make these updates within the submission form at the time of resubmission. Guidelines for resubmitting your figure files are available below the reviewer comments at the end of this letter. As the corresponding author, your ORCID iD is verified in the submission system and will appear in the published article. PLOS supports the use of ORCID, and we encourage all coauthors to register for an ORCID iD and use it as well. Please encourage your coauthors to verify their ORCID iD within the submission system before final acceptance, as unverified ORCID iDs will not appear in the published article. Only the individual author can complete the verification step; PLOS staff cannot verify ORCID iDs on behalf of authors.We look forward to receiving your revised manuscript. Kind regards, Simon RaynerAcademic EditorPLOS Neglected Tropical Diseases Qu ChengSection EditorPLOS Neglected Tropical Diseases

Shaden Kamhawi

co-Editor-in-Chief

PLOS Neglected Tropical Diseases

orcid.org/0000-0003-4304-636XX

Paul Brindley

co-Editor-in-Chief

PLOS Neglected Tropical Diseases

orcid.org/0000-0003-1765-0002

**Additional Editor Comments (if provided):** Hello

i see you have submitted a significantly revised manuscript and a point by point response to the comments provided by reviewer 1. However, you haven't responded to the questions submitted by reviewer 2. In addition, it would be helpful if instead of summarizing the original comments use simple phrases, you could include the original comments from the reviewers in your response letter,  as this would improve readability and make it easier to follow the revisions and responses.

The comments from Reviewer 2 are copied below:

Estimates of dog population

The reliance on a projected baseline of 8,000 dogs using the WHO recommendation of 5:1 leaves the stated 74% coverage metric vulnerable to denominator inflation. Given that the study explicitly notes a lack of strategies for free-roaming dogs, the true population-level immunity may be lower.

It would be interesting to hear the authors perspective on on how future iterations of this campaign might realistically integrate low-cost, resource-limited monitoring tools to validate these population projections and ensure the 70% WHO herd immunity threshold is truly met.

Household sampling

A sample size of 200 households is logistically understandable and consistent with similar pilot-scale One Health evaluations in resource-limited urban environments (e.g., Lusaka). Nevertheless, could the authors clarify their sampling design. For example, did these 200 households represent a general population sample or were participants pre-screened for dog ownership, as this might significantly affect the precision of the reported vaccination and ownership metrics.

KII

The authors rely on 10 key informant interviews to evaluate the One Health integration of the campaign. However, while a sample of 10 can achieve saturation in a highly homogeneous group, couldn’t i argue that the sample is somewhat heterogeneous, i.e., spanning four distinct sectors (veterinary, public health, environmental health, and community leadership) with differing institutional goals, vocabularies, and mandates.?

With only 2 to 3 participants per sub-group, was thematic saturation reached within any single sector? Specifically, you state that “. Four recurrent themes emerged from the thematic analysis” but are themes 2 “Operational Challenges During Campaign Implementation” and 3 “Inaccessibility for Rural and Peri-Urban Communities” reported across all foour groups, or only one?

Age restriction on participants

The restriction of household questionnaires to adults >= 18 is standard and logistically sound regarding household decision-making and animal ownership logistics. However, given that children are commonly the most vulnerable demographic to rabies in sub-Saharan Africa it would be interesting to hear the authors thoughts on this – Does relying solely on adult-focused data obscure critical gaps in community risk-reduction and bite reporting in a particularly vulnerable sub group of the population?

could you also provide your response to these comments too, as they don't seem to be addressed in your revision

Thank you

**Reviewers' comments:** [NOTE: If reviewer comments were submitted as an attachment file, they will be attached to this email and accessible via the submission site. Please log into your account, locate the manuscript record, and check for the action link "View Attachments". If this link does not appear, there are no attachment files.]**Figure resubmission:** While revising your submission, we strongly recommend that you use PLOS’s NAAS tool (https://ngplosjournals.pagemajik.ai/artanalysis) to test your figure files. NAAS can convert your figure files to the TIFF file type and meet basic requirements (such as print size, resolution), or provide you with a report on issues that do not meet our requirements and that NAAS cannot fix.

After uploading your figures to PLOS’s NAAS tool - https://ngplosjournals.pagemajik.ai/artanalysis, NAAS will process the files provided and display the results in the "Uploaded Files" section of the page as the processing is complete. If the uploaded figures meet our requirements (or NAAS is able to fix the files to meet our requirements), the figure will be marked as "fixed" above. If NAAS is unable to fix the files, a red "failed" label will appear above. When NAAS has confirmed that the figure files meet our requirements, please download the file via the download option, and include these NAAS processed figure files when submitting your revised manuscript. **Reproducibility:**  To enhance the reproducibility of your results, we recommend that authors of applicable studies deposit laboratory protocols in protocols.io, where a protocol can be assigned its own identifier (DOI) such that it can be cited independently in the future. Additionally, PLOS ONE offers an option to publish peer-reviewed clinical study protocols. Read more information on sharing protocols at https://plos.org/protocols?utm_medium=editorial-email&utm_source=authorletters&utm_campaign=protocols

# Author comment: Author response to Decision Letter 2

**DOI:** 10.1371/journal.pntd.0014080.r004

7 Aug 2026

Attachment Submitted filename: Response.docx

# Editor report: Decision Letter 2

**DOI:** 10.1371/journal.pntd.0014080.r005

**Authors:** Simon Rayner, Qu Cheng

15 Sep 2026

Dear Mr Dwaah,

We are pleased to inform you that your manuscript 'Evaluation of mass dog vaccination campaign in Techiman, Ghana' has been provisionally accepted for publication in PLOS Neglected Tropical Diseases.

Before your manuscript can be formally accepted you will need to complete some formatting changes, which you will receive in a follow up email. A member of our team will be in touch with a set of requests.

Please note that your manuscript will not be scheduled for publication until you have made the required changes, so a swift response is appreciated.

IMPORTANT: The editorial review process is now complete. PLOS will only permit corrections to spelling, formatting or significant scientific errors from this point onwards. Requests for major changes, or any which affect the scientific understanding of your work, will cause delays to the publication date of your manuscript.

Should you, your institution's press office or the journal office choose to press release your paper, you will automatically be opted out of early publication. We ask that you notify us now if you or your institution is planning to press release the article. All press must be co-ordinated with PLOS.

Thank you again for supporting Open Access publishing; we are looking forward to publishing your work in PLOS Neglected Tropical Diseases.

Best regards,

Simon Rayner

Academic Editor

PLOS Neglected Tropical Diseases

Qu Cheng

Section Editor

PLOS Neglected Tropical Diseases

Shaden Kamhawi

co-Editor-in-Chief

PLOS Neglected Tropical Diseases

orcid.org/0000-0003-4304-636XX

Paul Brindley

co-Editor-in-Chief

PLOS Neglected Tropical Diseases

orcid.org/0000-0003-1765-0002

***********************************************************

thank you for addressing the responses raised in previous version of the manuscript

# Editor report: Acceptance letter

**DOI:** 10.1371/journal.pntd.0014080.r006

**Authors:** Simon Rayner, Qu Cheng

Dear Mr Dwaah,

We are delighted to inform you that your manuscript, "Evaluation of mass dog vaccination campaign in Techiman, Ghana," has been formally accepted for publication in PLOS Neglected Tropical Diseases.

We have now passed your article onto the PLOS Production Department who will complete the rest of the publication process. All authors will receive a confirmation email upon publication.

The corresponding author will soon be receiving a typeset proof for review, to ensure errors have not been introduced during production. Please review the PDF proof of your manuscript carefully, as this is the last chance to correct any scientific or type-setting errors. Please note that major changes, or those which affect the scientific understanding of the work, will likely cause delays to the publication date of your manuscript. Note: Proofs for Front Matter articles (Editorial, Viewpoint, Symposium, Review, etc...) are generated on a different schedule and may not be made available as quickly.

Soon after your final files are uploaded, the early version of your manuscript will be published online unless you opted out of this process. The date of the early version will be your article's publication date. The final article will be published to the same URL, and all versions of the paper will be accessible to readers.

For Research Articles, you will receive an invoice from PLOS for your publication fee after your manuscript has reached the completed accept phase. If you receive an email requesting payment before acceptance or for any other service, this may be a phishing scheme. Learn how to identify phishing emails and protect your accounts at https://explore.plos.org/phishing.

Thank you again for supporting open-access publishing; we are looking forward to publishing your work in PLOS Neglected Tropical Diseases.

Best regards,

Shaden Kamhawi

co-Editor-in-Chief

PLOS Neglected Tropical Diseases

Paul Brindley

co-Editor-in-Chief

PLOS Neglected Tropical Diseases
